# Assessment of Female Pelvic Pathologies: A Cross-Sectional Study Among Patients Undergoing Magnetic Resonance Imaging for Pelvic Assessment at the Maternity and Children Hospital, Qassim Region, Saudi Arabia

**DOI:** 10.7759/cureus.46621

**Published:** 2023-10-07

**Authors:** Ziyad A Almushayti, Ibrahem A AlWahhabi, Rayan S Alkhedhairi, Abdulaziz S Alwahhabi, Faisal A Alzaidi, Sulaiman S Alsawyan, Mahmoud A Kasem

**Affiliations:** 1 Department of Radiology, College of Medicine, Qassim University, Al-Qassim, SAU; 2 College of Medicine, Qassim University, Al-Qassim, SAU; 3 Department of Radiology, Maternity & Children Hospital, Ministry of Health, Buraydah, SAU

**Keywords:** gynecology, endometriomas, dermoid cyst, uterine fibroids, magnetic resonance imaging, female pelvic pathologies

## Abstract

Background and objectives

Pelvic pathologies affect females in all age groups. They vary in size and location and can be wide in classification, such as masses, ectopic pregnancy, ovarian torsion, and ruptured ovarian follicles. Patients commonly present with gynecological complaints such as menstrual irregularities, abnormal pelvic bleeding, and infertility. Extra-gynecological symptoms such as dysuria and painful defecation can also manifest. To diagnose these pathologies, magnetic resonance imaging (MRI) and other imaging modalities can be useful alongside history and physical examination for early clinical diagnosis. Due to the importance of prevalence rate in predicting pathologies in a certain age and due to the lack of research studies on pelvic MRI studies in Qassim region, Saudi Arabia, this study aimed to demonstrate the wide spectrum of female pelvic pathologies that can be diagnosed using MRI in Qassim region, Saudi Arabia.

Methods and results

A cross-sectional study was conducted among patients referred to the MRI Department for evaluation of female pelvic pathologies at the Department of Radiology at Maternity and Children Hospital in Buraydah, Qassim region, Saudi Arabia. A total of 325 patients were included in the study, with the majority being in the age group of 31-40 years. Fibroids were the most common pathology, being present in more than one-fifth of the study sample, followed by neoplastic growths and placental pathologies. Inflammatory pathologies were the least common pathologies, being present in approximately 5% of the participants. Statistically significant associations were found between the age groups, and the presence of anomalies (p = 0.009), existence of neoplastic changes (p < 0.001), presence of placental pathologies (p < 0.001), inflammatory changes (p = 0.025), and adenomyosis (p = 0.028).

Conclusion

MRI data offer important new information about the prevalence of various disorders among different age groups in the Qassim region of Saudi Arabia. Younger age groups had much higher rates of anomalies, whereas older age groups had much lower rates. Adenomyosis and neoplastic alterations were more prevalent in the later age groups, but endometrioma was more prevalent in younger age groups. Placental pathologies were more prevalent in women in their middle years, while scar pregnancy was more prevalent in women between 31 and 40 years of age. Younger people, especially those between 16 and 20 years of age, were more likely to experience inflammatory alterations. In the younger age group, there was no discernible association between age and the prevalence of normal outcomes. These findings help us understand how different illnesses manifest differently as we get older and emphasize the value of taking aging into account when diagnosing and treating disorders.

## Introduction

In the pelvis are located urogenital and gastrointestinal organs, and pelvic pathologies affect females in all different age groups. The pathologies vary in size, location, and classification, such as masses, ectopic pregnancy, ovarian torsion, and ruptured ovarian follicles, to name a few. Patients can commonly present with gynecological complaints such as menstrual irregularities, abnormal pelvic bleeding, and infertility. Extra-gynecological symptoms such as dysuria and painful defecation can also manifest. To diagnose these pathologies, magnetic resonance imaging (MRI) and other imaging modalities can be useful alongside history and physical examination for early clinical diagnosis.

MRI is an excellent reproducible imaging technique that can be used in pregnant patients with suspicious abdominal or pelvic cancer. It does not use ionizing radiation and is generally considered safe for the fetus.

MRI use in gynecology provides a more detailed view of the female pelvic anatomy compared to other imaging modalities such as ultrasound and computed tomography (CT). It provides the option of utilizing a paramagnetic contrast agent and permits the capture of multiplanar images with high resolution without radiation exposure.

A more thorough assessment of the distribution and activity of the disease is possible in oncology owing to the morphological data that MRI may provide, such as size, contours, the number of lesions, edema, necrosis, and relationships to nearby structures [1].

MRI is also beneficial in the diagnosis of focal uterine lesions such as leiomyomas, diffuse disorders such as adenomyosis, evaluation of complex pelvic masses, sonographically indeterminate adnexal lesions, and detection as well as the staging of gynecological malignancies. Additionally, MRI is helpful in postoperative monitoring, identifying tumor recurrence, and distinguishing it from residual scarring after surgery [2]. In addition, it is also considered generally safe for pregnant women since it does not use ionizing radiation, giving it an advantage over other imaging modalities [3]. Finally, MRI is also a helpful problem-solving tool in situations where US findings are not conclusive [4].

Despite the potential usefulness of MRI as a tool for inconclusive diagnostics, we have been unable to find any local literature specifically pertaining to the identification or prevalence of female pelvic pathologies using MRI. Thus, this study aims to identify the diverse array of female pelvic pathologies that can be detected and diagnosed through MRI imaging to give physicians the proper data for future conducted studies to use our data for analysis or comparison.

## Materials and methods

Study design

This was a cross-sectional study conducted at the Maternity and Children Hospital in Buraydah, Qassim region, Saudi Arabia. The duration of the study was six months, with a target sample size of 300 patients. The study was conducted among patients referred for evaluation of female pelvic pathologies to the Department of Radiology at Maternity and Children Hospital in Buraydah, Qassim region in Saudi Arabia, from June 1, 2022, to December 31, 2022. Patient data (age and diagnosis) were taken directly via the PACS system. Patients older than 16 years who underwent MRI examination and gave consent were included in the study. Patients younger than 16 years, those who did not undergo MRI examination, and those who did not consent to participate in the study were excluded from the study.

Pilot study

The investigational criteria have already been utilized in studies of a comparable kind; hence, no pilot research is required at this time.

Data management and analysis plan

To ensure that the sample truly reflects the population, the data collected from the Department of Radiology was given the appropriate weighting. The SPSS Version 25 (IBM Corp., Armonk, NY) was used to analyze the data. Percentages and frequencies were used to present categorical variables, while mean and standard deviation were used to present continuous variables. Pearson’s chi-square test (χ2) was used to assess the statistical association between age groups and measurements including anomalies, fibroids, dermoid cysts, endometrioma, neoplastic changes, adenomyosis, placental pathologies, scar pregnancy, inflammatory changes, and normal MRI outcomes. A p-value of less than 0.05 was considered to be considered statistically significant. Cramer's V was used to analyze the strength of association; a value of <0.20 denotes a weak association, 0.20-0.30 denotes a moderate association, and >0.30 denotes a high association.

Ethical considerations

The acquired data's confidentiality was upheld, and appropriate security measures were taken. There was no individual reporting of patient data, and any form of patient identifiers were removed from the final study.

## Results

Table 1 presents the demographic and clinical characteristics of the research population (n = 325). In terms of the ages of the people who took part in the study, the majority were between 31 and 40 years of age (approximately one-third of the study sample). This was followed by the age category of 41 to 50 years. A sizeable proportion of the study population (17%) fell within the age range of 21 to 30 years. In addition, only one-fifth of the participants were over the age of 50, whereas the smallest age group, 16-20 years, comprised only 16 (4.9%) participants.

**Table 1:** Demographic and clinical characteristics of the study population (n = 325)

Characteristics	Category	n	%
Age	16-20 years	16	4.9
	21-30 years	55	16.9
	31-40 years	101	31.1
	41-50 years	90	27.7
	Above 50 years	63	19.4
Pathologies	Anomaly	11	3.4
	Fibroids	85	26.2
	Dermoid cysts	28	8.6
	Endometrioma	24	7.4
	Neoplastic growth	65	20..0
	Adenomyosis	18	5.5
	Placenta	33	10.2
	Scar pregnancy	23	7.1
	Inflammatory changes	17	5.2
	Normal	21	6.5

**Figure 1:**
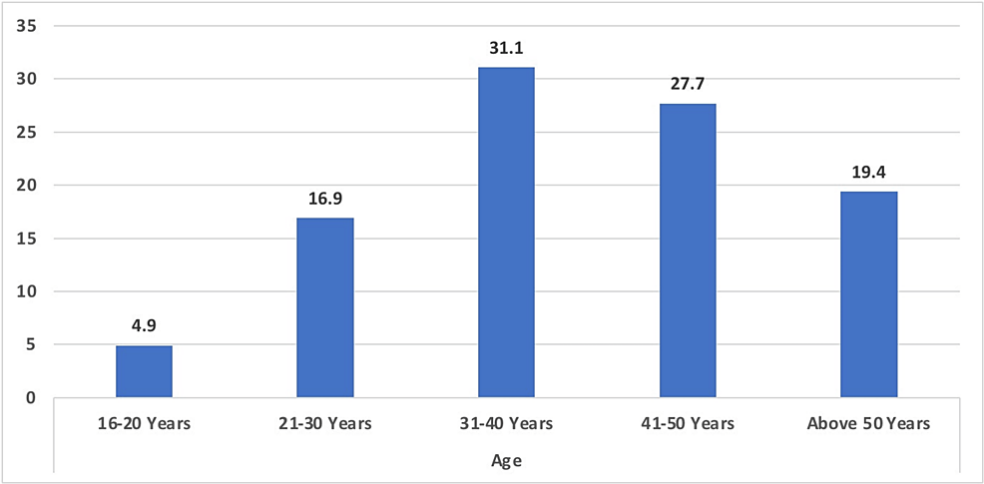
Graphical representation of the age of the study population

**Figure 2:**
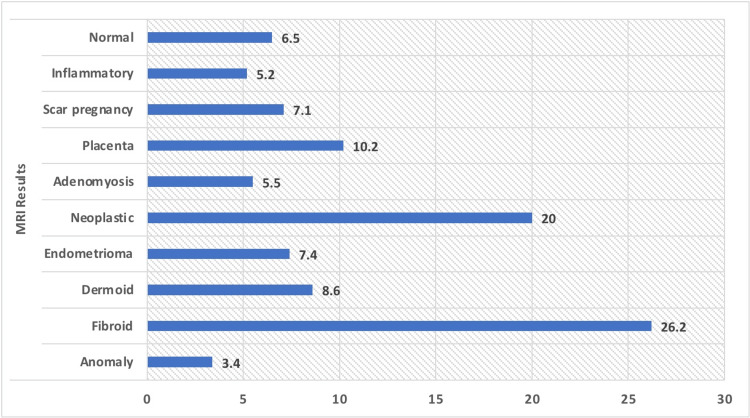
Graphical representation of the pathologies of the study population

Regarding pathologies, the research population revealed a variety of findings. The most prevalent anomaly detected was fibroids, which was present in more than 26% of the participants. In addition, more than 8% of the participants were found to have dermoid cysts, while more than 7% were found to have endometriomas. Neoplastic growths were observed in one-fifth of the study sample. Adenomyosis was observed in more than 5% of the participants. In addition, approximately 10% had abnormal placentas, and approximately 7% had scar pregnancies (Table 1 and Figures 1, 2).

The prevalence of anomalies was noted to be the lowest in people older than 50 years (0.0%) and highest in those between 21 and 30 years of age (10.9%). A moderate effect size (Cramer's V = 0.21) was found by a chi-square test between age group and the occurrence of anomalies (X^2^ = 13.61, p = 0.009). The prevalence of fibroids was lowest in those over the age of 50 years (17.9%) and highest in those between 31 and 40 years of age (30.7%). The existence of fibroids was not associated with age group (X^2^ = 4.36, p = 0.360). Similar to this, dermoid cysts were more common in participants aged 16 to 20 years (18.8%) than in those aged 41 to 50 years (5.6%). Dermoid cysts were present regardless of age group (X^2^ = 8.13, p = 0.087; Cramer's V = 0.16); however, there was no association between the two.

Those in the age group of 21 to 30 had the highest prevalence of endometrioma (12.7%), while those aged 50 years and above had the lowest prevalence (0%). However, there was no association between age group and the existence of endometrioma (X^2^ = 7.71, p = 0.103). Neoplastic alterations were most prevalent in people over 50 years of age (58.7%), and they were least prevalent in those between 31 and 40 years of age (5%). The results of the chi-square test showed a strong association (Cramer's V = 0.50) between age group and the existence of neoplastic changes with (X^2^ = 77.75, p < 0.001). Similarly, the prevalence of adenomyosis was zero in those under 16 years of age and 11.1% in those between 41 and 50 years of age. Age group and the presence of adenomyosis were associated, albeit insignificantly.

The prevalence of placental pathologies was highest in women between 31 and 40 years of age (21.8%) and lowest in those over the age of 50 years (1.6%). With a moderate effect size (Cramer's V = 0.27), a chi-square test showed a significant association between age group and the occurrence of placental pathologies (X^2^ = 24.00, p < 0.001). Scar pregnancy was most common in women aged 31 to 40 years (10.9%) and least common in women over 50 years of age (0.0%). There was a minor but statistically significant effect size (Cramer's V = 0.17) between age and the occurrence of scar pregnancy (X^2^ = 9.64, p = 0.047). Likewise, the prevalence of inflammatory alterations was highest in people between 16 and 20 years of age (18.8%) and lowest in those between years of age (1.0%). There was a significant association between age group and the presence of inflammatory changes (X^2^ = 11.18, p = 0.025, Cramer's V = 0.19). Normal results were most prevalent in the age group of 16-20 years (12.5%) and least prevalent in those older than 50 years (1.6%). There was no association between age group and normal results (X^2^ = 4.63, p = 0.328). Table 2 shows details.

**Table 2:** Distribution of responses among age groups in terms of MRI results (n = 325)

Pathologies	Response	Age (years)					Χ^2^	P-value	Cramer’s V
		16-20	21-30	31-40	41-50	Above 50			
Anomaly	No	15 (93.8)	49 (89.1)	98 (97.0)	89 (98.9)	63 (100.0)	13.61	0.009	0.21
	Yes	1 (6.3)	6 (10.9)	3 (3.0)	1 (1.1)	0 (0.0)			
Fibroids	No	13 (81.3)	41 (74.5)	70 (69.3)	64 (71.1)	52 (82.5)	4.36	0.360	0.12
	Yes	3 (18.8)	14 (25.5)	31 (30.7)	26 (28.9)	11 (17.5)			
Dermoid cysts	No	13 (81.3)	46 (83.6)	94 (93.1)	85 (94.4)	59 (93.7)	8.13	0.087	0.16
	Yes	3 (18.8)	9 (16.4)	7 (6.9)	5 (5.6)	4 (6.3)			
Endometrioma	No	15 (93.8)	48 (87.3)	92 (91.1)	83 (92.2)	63 (100.0)	7.71	0.103	0.15
	Yes	1 (6.3)	7 (12.7)	9 (8.9)	7 (7.8)	0 (0.0)			
Neoplastic growth	No	13 (81.3)	51 (92.7)	95 (94.1)	75 (83.3)	26 (41.3)	77.75		0.49
	Yes	3 (18.8)	4 (7.3)	6 (5.9)	15 (16.7)	37 (58.7)			
Adenomyosis	No	16 (100.0)	54 (98.2)	99 (98.0)	80 (88.9)	58 (92.1)	10.87	0.028	0.18
	Yes	0 (0.0)	1 (1.8)	2 (2.0)	10 (11.1)	5 (7.9)			
Placenta	No	16 (100.0)	50 (90.9)	79 (78.2)	85 (94.4)	62 (98.4)	24.00		0.27
	Yes	0 (0.0)	5 (9.1)	22 (21.8)	5 (5.6)	1 (1.6)			
Scar pregnancy	No	16 (100.0)	52 (94.5)	90 (89.1)	81 (90.0)	63 (100.0)	9.64	0.047	0.17
	Yes	0 (0.0)	3 (5.5)	11 (10.9)	9 (10.0)	0 (0.0)			
Inflammatory changes	No	13 (81.3)	53 (96.4)	100 (99.0)	83 (92.2)	59 (93.7)	11.18	0.025	0.19
	Yes	3 (18.8)	2 (3.6)	1 (1.0)	7 (7.8)	4 (6.3)			
Normal	No	14 (87.5)	51 (92.7)	92 (91.1)	85 (94.4)	62 (98.4)	4.63	0.328	0.12
	Yes	2 (12.5)	4 (7.3)	9 (8.9)	5 (5.6)	1 (1.6)			

## Discussion

Using MRI results, the present study investigated the presence of various pathologies in distinct age groups in the Qassim region of Saudi Arabia. In various age groups, the prevalence of anomalies, fibroids, and dermoid cysts exhibited distinct patterns. The significance and originality of these results can be determined by comparing them to prior research. It was discovered that anomalies were most prevalent in the age group of 21 to 30 years, while they were wholly absent in those older than 50 years. According to past literature [5], abnormalities are more common in younger people. The results of our study are in accordance with the literature. For a variety of causes, including genetic predisposition, aberrant developmental patterns, and early environmental impacts, anomalies are more prevalent in younger age groups. The lack of anomalies in individuals over the age of 50 years may imply that some anomalies diminish with aging and become more difficult to identify, or that individuals with major anomalies may not live to older ages. To completely comprehend the underlying mechanisms and long-term ramifications of these discoveries, future studies may be warranted.

Fibroid prevalence was shown to be highest in women between 31 and 40 years of age, with a subsequent decline in incidence among women aged 50 years and above. Although age and the prevalence of fibroids did not correlate statistically significantly, earlier research [6] has shown that fibroids are more common in women between 31 and 40 years of age. Progesterone and estrogen both have crucial roles in the development and growth of fibroid tumors [7,8]. The higher occurrence among people between 31 and 40 years of age may be related to the hormonal changes that occur throughout the reproductive years. The significance of other risk factors, such as hormonal imbalances, ethnicity, and genetic predisposition, which have all been extensively discussed in the literature, is not diminished by the fact that there was no significant association between age group and fibroids in this study [9].

The prevalence of dermoid cysts was similar; it peaked between 16 and 20 years of age before declining between 41 and 50 years of age. The pattern observed is consistent with other research that indicated younger adults to have a higher prevalence of dermoid cysts [10], even though there was no statistically significant association between age group and dermoid cysts. According to prior studies [11], dermoid cysts are frequently congenital and are a result of abnormal germ cell migration during embryonic development. The natural regression of these cysts over time or early diagnosis and excision of these cysts could be reasons for the decline in prevalence with age. There is a need to carry out further research to ascertain the underlying mechanisms and therapeutic implications.

Endometrioma was most prevalent in those between 21 and 30 years of age, whereas cases were not found in people over the age of 50 years. The pattern of a higher prevalence in younger age groups is consistent with earlier studies [12], despite the lack of a statistically significant association between age and endometrioma. According to Brosens et al. [13], endometrioma typically affects fertile females and is linked to immunological and hormonal variables. The alterations in hormones due to menopause and the subsequent regression of endometriotic lesions may be the cause of the absence of endometrioma in women over the age of 50 years.

The prevalence of neoplastic alterations was highest in people over 50 years of age and lowest in those between 31 and 40 years. There is a strong association between age and the prevalence of neoplastic changes, as demonstrated by earlier research that found the incidence of neoplasms to increase with aging [14]. According to previous studies [15], age is an established risk factor for a variety of neoplastic conditions, including ovarian, uterine, and cervical malignancies. The cumulative exposure to environmental factors, genetic mutations, and hormone effects over time may explain why neoplastic alterations are more common in people over 50 years of age. The modest effect size, which demonstrates a strong correlation between age group and neoplastic changes, suggests that age should be taken into consideration while diagnosing and treating these illnesses.

Additionally, the prevalence of adenomyosis was higher in those aged 41 to 50 years, but not in those between 16 and 20 years of age. The substantial correlation between age and the existence of adenomyosis is in line with earlier studies showing that elderly women are more likely to have the disorder than younger women [16]. Adenomyosis is defined by the presence of endometrial tissue within the myometrium and is usually linked to dysmenorrhea and infertility [17]. The increasing incidence among women between 41 and 50 years of age may be explained by the cumulative effect of estrogen exposure and changes in the uterine microenvironment over time. The poor association between age group and adenomyosis implied by the tiny effect size increases the possibility that hormonal variables and genetic predisposition may potentially play a role in the onset of adenomyosis.

The prevalence of placental pathologies was much lower among women over 50 years of age and was highest among those between 31 and 40 years of age. This finding raises the possibility that the placenta and the middle-aged population are related. Although age was a contributing factor and there was a statistically significant association between age group and placental pathologies, pathologies associated with the placenta are frequently seen during pregnancy and may have effects on the health of both the mother and the baby [18]. The higher occurrence in this age range may be explained by the higher chance of pregnancy among women aged 31 to 40 years. However, further research is required to look into the underlying mechanisms and potential therapeutic applications of these findings due to the impact magnitude's relatively small size.

Women above the age of 50 years did not have scar pregnancy; however, women between the ages of 31 and 40 experienced it most frequently. The association between age group and scar pregnancy was statistically significant despite the small impact size. Scar pregnancies are uncommon but dangerous complications that occur when a pregnancy implants within a uterine scar from a previous cesarean section or another surgical treatment [19]. The increased risk of prior cesarean sections or other uterine surgeries may account for the higher incidence in women between 31 and 40 years of age. Due to a reduced prevalence of recent surgical procedures, women over 50 years of age may not have scar pregnancies.

The age group of 16 to 20 years had the highest prevalence of inflammatory changes, whereas the age group of 31 to 40 years had the biggest decrease. Age group and inflammatory changes were associated with a modest statistical value. Infections, trauma, underlying inflammatory illnesses, and other factors can cause inflammatory alterations in the pelvis [20]. There may be a greater propensity for infections and risky sexual behavior among people between 16 and 20 years of age that accounts for the higher incidence. Older adults may be less prone to infections or less likely to engage in high-risk behaviors than younger people, which may account for the reduction in prevalence within this age range.

Although the association between age group and normal results was not statistically significant, normal results were more common in the age group of 16-20 years and less common in those over 50 years of age. The effect's small size suggests that there is little association between age group and typical outcomes. The lack of diseases or abnormalities in the imaged region is shown by normal MRI results. Younger age groups tend to have a larger prevalence of normal outcomes, which may be attributed to a healthier physiological state and a lower prevalence of age-related diseases. The lack of a meaningful correlation, however, shows that factors other than age alone need to be taken into account when interpreting normal MRI results.

Limitations

This study has some limitations that should be acknowledged. First of all, the study's applicability to other groups or locations may be limited because it was only conducted in the Qassim region of Saudi Arabia. The results of the study were also based on MRI scans, which may have inherent limitations when it comes to diagnosing particular disorders or detecting minute changes. The study's cross-sectional nature prevents it from understanding the temporal associations between age and diseases and establishing causation. Longitudinal studies would be helpful in examining the dynamic shifts in these illnesses' prevalence across different age groups. These disorders may also be influenced by pertinent factors that were not examined in this study, such as hormone levels, dietary practices, and genetic predisposition. To develop a more thorough understanding of the intricate origins and effects of various illnesses, future research should take these factors into account.

Implications

Numerous implications for clinical practice and ongoing research flow from this study's findings. First, by accounting for age-related differences in a disease's prevalence, medical experts can more precisely assess risk, make diagnoses, and formulate treatment plans. The creation of screening and intervention strategies that are suitable for particular age groups can be aided by an understanding of the prevalence and patterns of different illnesses according to age. The study also emphasizes the necessity for more investigation into the mechanisms and constituents of these aging-related behaviors. If examined, genetic predisposition, interactions between aging and hormones, and other pertinent elements may help us understand how these diseases develop and spread. The results highlight the need to consider age as a crucial factor in research and clinical practice in order to enhance the precision of diagnosis, prognosis, and treatment outcomes for individuals with these disorders.

## Conclusions

This study draws the conclusion that the MRI data offer important new information about the prevalence of various disorders among different age groups in Saudi Arabia. The results corroborate the findings' uniqueness and significance in terms of the occurrence of specific disorders in various age groups. Younger age groups saw much higher rates of anomalies, whereas older age groups saw much lower rates. Adenomyosis and neoplastic alterations were more prevalent in later age groups, but endometrioma was more prevalent in younger age groups. Placental pathologies were more prevalent in women in their middle years, while scar pregnancy was more prevalent in women between 31 and 40 years of age. Younger participants, especially those between 16 and 20 years of age, were more likely to experience inflammatory alterations. These findings help us understand how different illnesses manifest differently as we get older and emphasize the value of taking aging into account when diagnosing and treating disorders.
